# Relationships Between Resilience, Mental Well-Being, and COVID-19 Worries in Collegiate Student-Athletes

**DOI:** 10.3389/fspor.2022.890006

**Published:** 2022-05-11

**Authors:** Cade J. Watts, Robert C. Hilliard, Scott Graupensperger

**Affiliations:** ^1^Department of Exercise Science, Shenandoah University, Winchester, VA, United States; ^2^Department of Psychiatry and Behavioral Sciences, University of Washington, Seattle, WA, United States

**Keywords:** mental health, flourishing, COVID-19 anxiety, resilience, well-being, Division III

## Abstract

The onset of the COVID-19 pandemic was associated with robust declines in well-being for collegiate student-athletes. Worries about COVID-19 have frequently been associated with worsening well-being; therefore, it is important to examine protective factors against well-being decrements. Resilience, one's ability to respond to stress and adversity, may be one such factor. Despite this possible influence, resilience has not yet been studied in student-athletes in this context as the pandemic has progressed. Therefore, the purpose of this study was to examine the moderating role of resilience on the relationship between COVID-19 worries and well-being. In this cross-sectional design, National Collegiate Athletic Association Division III athletes (*N* = 91) at one university completed surveys on COVID-19 worries, resilience, and well-being between February and March 2021. All competitions had been postponed until the Spring 2021 semester. The findings revealed a negative correlation between COVID-19 worries and well-being (*r* = −0.21, *p* = 0.05) and a positive correlation between resilience and well-being (*r* = 0.44, *p* < 0.001). Additionally, multiple regression and simple slopes analyses showed that individuals with higher resilience endorsed greater scores of well-being, even when COVID-19 worries increased (β = 0.38, *p* = 0.02). In conclusion, our results suggest that resilience had a moderating effect on the relationship between COVID-19 worries and well-being.

## Introduction

On March 11th, 2020, the World Health Organization recognized the coronavirus disease of 2019 (COVID-19) as a pandemic (World Health Organization., 2020). Following the WHO's position, many countries entered a state of national lockdown to help prevent further spread of COVID-19. Unfortunately, the lockdowns and related stressors led to worsening mental well-being (MWB) in general populations across the world (Ammar et al., 2020b; Graupensperger et al., 2020; Xiong et al., 2020). MWB can have many definitions, but here we define it using the flourishing construct (Diener et al., 2010), which is based on humanistic psychology theories and measures psychological prosperity. Most studies have focused on negative aspects of MWB, despite the many definitions considering positive daily functioning (Schary and Lundqvist, 2021).

For example, Newby et al. (2020) found that, early in the pandemic, 78% of Australians reported a worsening of MWB. Furthermore, based on combined data ranging from early February 2020 to early July 2020 from several countries across the globe, prevalence of depressive symptoms ranged from 15.1 to 72.6% (Guo et al., 2020; Makhashvili et al., 2020; Newby et al., 2020; Passos et al., 2020; Vujčić et al., 2021). Anxiety prevalence, based on the aforementioned parameters, was ~20.2 to 71.3% (Makhashvili et al., 2020; Newby et al., 2020; Passos et al., 2020; Vujčić et al., 2021). Although studied less frequently, similar decreases were found when measuring flourishing (e.g., Kavčič et al., 2021; Sürücü et al., 2021; VanderWeele et al., 2021).

College students were not exempt from these decrements, as several studies reported increased levels of anxiety, depression, and need for help-seeking early on in the pandemic (e.g., Huckins et al., 2020; Kecojevic et al., 2020; Browning et al., 2021; Fruehwirth et al., 2021; Jones et al., 2021), as well as reduced flourishing (Nyunt et al., 2022). Included in this sample is student-athletes, who experienced immediate and potential long-term impacts on MWB from the pandemic (Bullard, 2020; National Collegiate Athletic Association., 2020; Valster et al., 2021).

For student-athletes, one of the immediate consequences of the COVID-19 related lockdowns were cancellations of sports seasons. As such, removal from sport limited teammate interactions, which are a hallmark of student-athlete social support groups. The disruptions to these social groups may adversely affect MWB in these individuals and thereby lead to other negative mental health outcomes (Ammar et al., 2020a; Graupensperger et al., 2020). Initial data collected after the onset of lockdowns suggested decreases in student-athlete MWB (National Collegiate Athletic Association., 2020). Therefore, based on a recent model (Paredes et al., 2021), this study aimed to examine two potential factors that could be associated with MWB in collegiate student-athletes: COVID-19 worries and resilience.

## COVID-19 Worries and Well-Being

It is clear that the pandemic had a detrimental effect on MWB in the general population (e.g., Ammar et al., 2020b; Guo et al., 2020; Browning et al., 2021). However, minimal research has been published observing the effect of COVID-19 on collegiate student-athletes' MWB throughout the pandemic. Current evidence indicates that COVID-19 acts as a negative stressor for athletes which may contribute to MWB deficits (Haan et al., 2021). These data are supported by National Collegiate Athletic Association (National Collegiate Athletic Association., 2020, 2021) cross sectional data from the Spring and Fall 2020 semesters. At pandemic onset, some mental health concerns increased by 200% compared to pre-pandemic levels, with reports of anxiety and feeling overwhelmed ranging from 11 to 50% (National Collegiate Athletic Association., 2020). Of particular concern is the lack of significant change in many facets of MWB compared to the same survey that was conducted in Spring 2020 (National Collegiate Athletic Association., 2021). In fact, student-athletes at one NCAA Division III university reported distress increased throughout the Fall 2020 semester (Valster et al., 2021). Taken together, these data suggest that even as the pandemic continues, there is still a need to explore MWB levels in student-athletes.

There are several possible explanations for why MWB might have decreased during the pandemic, but a consistent set of constructs associated with negative changes are worries about COVID-19. In the context of COVID-19, these worried thoughts in the general population may extend to uncertainty about the future, financial security, social isolation, and, in particular, worry of COVID-19 infection (Liu et al., 2020; Makhashvili et al., 2020; Newby et al., 2020; Vujčić et al., 2021). Some studies suggest that college students have a greater prevalence of COVID-19-specific worries when compared to the general population (Newby et al., 2020; Xiong et al., 2020; Vujčić et al., 2021). Increased worries in college students may be influenced by increased concern for academic performance (Kecojevic et al., 2020; Son et al., 2020; Wang et al., 2020; Browning et al., 2021).

Regarding student-athletes, less is known about their levels of worries. Graupensperger et al. (2020) reported an average score of 3.46 (out of 5) for COVID-19 worries at the onset of the pandemic, but Sanborn et al. (2021) reported NCAA Division I student-athletes had very low COVID-19 anxiety and minimal psychological symptoms. Regardless of current levels, given the clear association of these worries on MWB, it is prudent to continue examining factors that promote MWB.

## Resilience and Well-Being

It is clear student-athletes are at risk for diminished MWB as a result of COVID-19. One possible construct that could act as a protective factor for MWB is resilience. Resilience is essentially a measure of an individual's ability to cope with adversity and be protected from the negative effects of stressors (Connor and Davidson, 2003; Sarkar and Fletcher, 2014). Given that the pandemic has resulted in numerous stressors and a need for adaptation, resilience should theoretically help ameliorate decreases in MWB.

Current evidence in non-athlete populations supports this theoretical assertion. Researchers have found a positive relationship between MWB and resilience (e.g., Killgore et al., 2020; Knowles et al., 2021; Kocjan et al., 2021). A large-scale review of MWB during the pandemic also found that generally, more resilient individuals were faring more positively (Manchia et al., 2022). Importantly, resilience has substantially reduced or eliminated the effect of various COVID-19 concerns on MWB (Chan et al., 2021; Kavčič et al. 2021; Kocjan et al., 2021; Paredes et al., 2021; Shah et al., 2022).

Little of this resilience research has been conducted with athletes, but the studies with these populations support these findings (e.g., Gupta and McCarthy, 2021; Knowles et al., 2021). Studies suggest loss of social connectedness amongst teammates following the COVID-19 pandemic was positively correlated with decreased MWB (Graupensperger et al., 2020) and that even when COVID-19 anxiety is low, it is still correlated to depression (Sanborn et al., 2021). Athletes did not appear to frequently seek counseling services for their distress when the pandemic began (Slavin et al., 2022), so understanding traits that might help them cope if they are not seeking services will benefit their MWB. The evidence suggests that resilience might be one of these factors, and the trait becomes even more valuable to study in student-athletes when considering the role resilience plays in performance (Sarkar and Fletcher, 2014).

## The Current Study

Despite some of this previous knowledge, there are still gaps in the literature. When examining general MWB and mental health during the pandemic, most studies, especially those focused on student-athletes, have emphasized negative mental health outcomes (Schary and Lundqvist, 2021). Currently, more is known about the negative effects on MWB from the pandemic in student-athletes (e.g., Bullard, 2020; National Collegiate Athletic Association., 2020, 2021; Slavin et al., 2022). It is important that we also learn more about how student-athletes are experiencing positive psychological functioning in their lives to get a clearer picture of the effects of the pandemic (Schary and Lundqvist, 2021). Additionally, little is known about the MWB of DIII student-athletes during the pandemic outside of a few studies (e.g., Bullard, 2020; National Collegiate Athletic Association., 2020, 2021; Valster et al., 2021). Most of these studies were conducted early on in the pandemic, and researchers who did examine college-students in the Fall 2020 semester found increased stress through the semester (Valster et al., 2021) and flourishing levels still lower than pre-pandemic (Nyunt et al., 2022). Therefore, the Spring 2021 semester not only provided a snapshot of the continued effects of the pandemic, but was a semester that provided unique challenges for many DIII athletes.

After returning to campus, collegiate student-athletes were subject to rigorous and repeated COVID-19 testing, quarantine following suspected close-contact to a positive COVID-19 case, and isolation for 14 days following a positive COVID-19 diagnosis.

For the 2020–2021 school year, the Old Dominion Athletic Conference postponed all sports until the Spring 2021 semester. Most athletes experienced pre-determined shortened seasons, and COVID-19 outbreaks often resulted in team shutdowns or further cancellation of games. Student-athletes in our study were subjected to rigorous and repeated COVID-19 testing, quarantine following suspected close-contact to a positive COVID-19 case, and isolation for 14 days following a positive COVID-19 diagnosis. Adding to this stress, DIII colleges and universities do not have as many athletic facilities when compared to DI schools. Therefore, when all conference DIII sports were played in the spring season, added stressors related to practice spaces, strength and conditioning, and game facility shortages occurred. Altogether, the unique context of the Spring 2021 semester and evidence that distress was still rising in student-athletes (Valster et al., 2021) necessitated further study of DIII student-athletes' MWB and the potential role of resilience in this process.

Therefore, the aim of this study was to identify the association of COVID-19 worry with MWB and determine if resilience moderates that relationship. Specifically, we hypothesized that: (1) COVID-19 worries would be negatively associated with MWB, (2) resilience would be positively associated with MWB, and (3) resilience would moderate the relationship between COVID-19 worries and MWB such that the adverse relationship of COVID-19 worries on MWB would be weaker for athletes higher in resilience.

## Materials and Methods

### Participants

To be eligible for this study, all participants were required to be at least 18 years of age (*M* = 19.8, *SD* = 1.2) and participate in NCAA DIII varsity sports. No participants were excluded from the study if they met eligibility requirements. Initially, 97 participants completed the online questionnaires. However, due to a technological error, the first six participants were unable to complete the resilience measure. Therefore, the final sample consisted of 91 participants (N_female_ = 54). Overall, student-athletes were recruited from 11 sports at a single institution and were between 18 and 23 years of age (*M* = 19.8, *SD* = 1.2). The sample was 77% White and spread amongst first-year (*n* = 29), sophomore (*n* = 21), junior (*n* = 23), senior (*n* = 14), and graduate (*n* = 4) students.

### Measures

#### Resilience

Resilience was measured using the Connor-Davidson Resilience Scale 10 (CD-RISC 10; Campbell-Sills and Stein, 2007). This scale uses 10 items from the original 25-item scale (1, 4, 6, 7, 8, 11, 14, 16, 17, 19), with scores ranging from 0 to 40 (Campbell-Sills and Stein, 2007). Participants rate a variety of statements on a 5-point Likert scale ranging from 0 (*not true at all*) to 4 (*true nearly all the time*). Higher scores indicate higher resilience. The 10-item variation is reliable (Cronbach's *a* = 0.85) and valid (Campbell-Sills and Stein, 2007). Cronbach's α = 0.85 in the current study.

#### Mental Well-Being

MWB was assessed using the Flourishing Scale (Diener et al., 2010). This 8-item scale has participants respond to each statement on a 7-point Likert scale ranging from 1 (*strongly disagree*) to 7 (*strongly agree*). A sample item is “I live a purposeful and meaningful life*.”* Higher scores indicate more flourishing. Validation testing was conducted on international, geographically diverse, college students and is reliable (Cronbach's *a* = 0.87) and temporally stable (0.71). The Flourishing Scale has high convergent validity and was strongly correlated with similar well-being scales (Diener et al., 2010). The Cronbach's *a* = *0.9*1 in this sample.

#### COVID-19 Worries

We measured COVID-19 worries using a 4-item scale designed for suggested use in the pandemic (Graupensperger et al., 2020; Hensel et al., 2021). This scale uses a 5-point Likert scale ranging from 1 (*does not apply at all*) to 5 (*strongly applies*) to gauge participant responses to: “I am nervous when I think about current circumstances,” “I am worried about my health,” “I am worried about the health of my family members,” and “I feel stressed about leaving my house.” This scale was highly reliable (Cronbach's *a* = 0.81) in a sample of college student-athletes (Graupensperger et al., 2020) and demonstrated similar reliability in our sample (Cronbach's *a* = 0.77).

#### Demographics

All participants provided information on age, gender, race, sport, academic year, and whether or not they ever received a positive COVID-19 diagnosis.

### Procedure

After receiving approval from the Shenandoah University institutional review board, the first author sent emails to head coaches of every sports team (22) at the university. The emails informed coaches about the nature of the study and requested that they disseminated the study to their respective student-athletes. Prospective participants received relevant information about the study, including the survey link and informed consent document, which participants completed before beginning the study. The survey stayed open for 2 weeks between February and March 2021. This survey was conducted using a Google Form restricted to the institution. The participants did not receive compensation.

### Analytic Strategy

Preliminary analyses entailed estimating bivariate correlations between study variables. To test the primary study aims, multiple linear regression models were fit iteratively to assess main effects separately from interaction effects. In the first model, the main effect of COVID-19 worries on MWB was estimated. The second model added resilience as a covariate and, finally, the third model estimated the interaction between COVID-19 worries and resilience to test the hypothesis that resilience would moderate the association between COVID-19 worries and MWB. To further probe the interaction estimated in model 3 we plotted the interaction and estimated simple slopes of the association between COVID-19 worries and MWB at +/-1 standard deviation on resilience (i.e., interpreted as those relatively high/low in resilience). Regression models controlled for participants' age, gender (0 = female; 1 = male), race/ethnicity (0 = White; 1 = other), and year in school. Analytic models were estimated in R software. All alphas were set at 0.05 to determine statistical significance.

## Results

Excluding the previously described missing data for six participants on the CD-RISC, there was no other missing data. A preliminary screening suggested normally distributed data that met all assumptions for multiple regression analyses.

Descriptive statistics and bivariate correlation coefficients are shown in Table 1. With alpha set to 0.05 for statistical significance, older athletes reported greater MWB, on average. MWB was inversely correlated with COVID-19 worry and positively correlated with resilience, whereas resilience and COVID-19 worry were inversely related.

**Table 1:** Descriptive statistics and bivariate correlation estimates (*N* = 91).

	**1**	**2**	**3**	**4**	**5**
1. Age	–				
2. Year in School	0.85^***^	–			
3. Flourishing	0.19	0.27^**^	–		
4. COVID-19 worries	−0.04	0.01	−0.21^*^	–	
5. Resilience	0.14	0.06	0.44^***^	−0.49^***^	–
Mean	19.78	-	46.80	11.14	28.77
SD	1.15	-	7.55	3.33	6.14
Range	18–22	-	8–56	5–20	0–40

* *p < 0.05*,

** *p < 0.01*,

*** *p < 0.001*.

**Figure 1:**
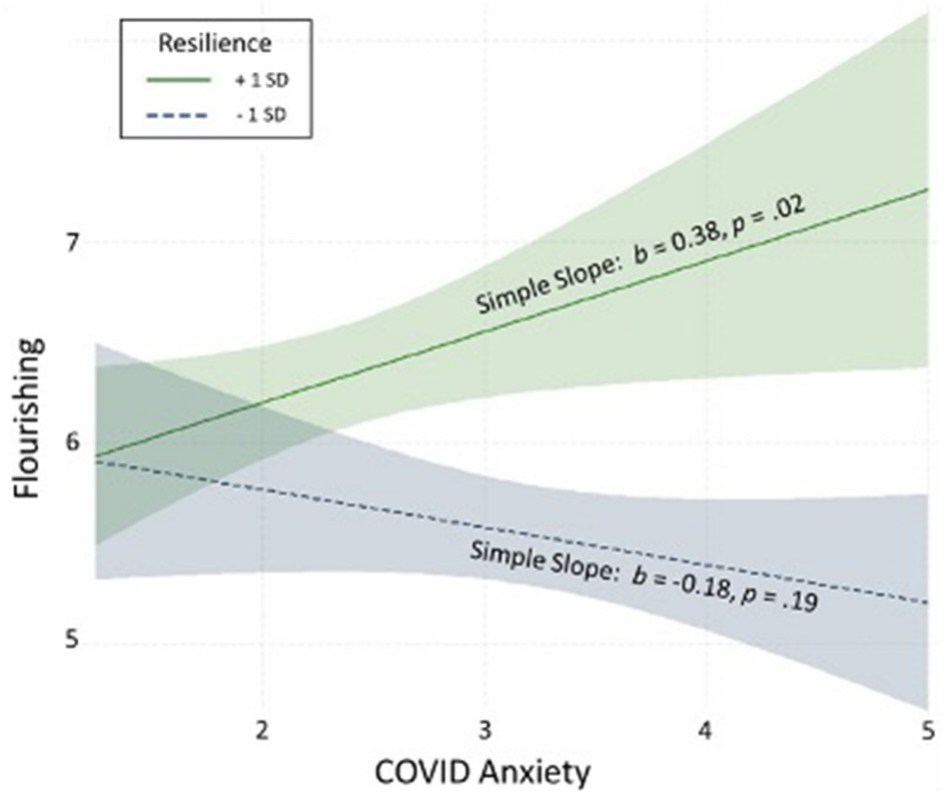
Simple slopes decomposing the moderating effect of resilience on the association between COVID anxiety and flourishing.

The primary results, multiple linear regression models, are displayed in Table 2. Holding control variables constant, COVID-19 worries inversely related to MWB, though this effect was not quite statistically significant (Model 1). As shown in Model 2, resilience was significantly related to higher MWB scores, and adding this covariate explained an additional 15% of variance in MWB. As it pertains to the interaction between COVID-19 worries and resilience, Model 3 revealed that resilience significantly moderated the effect of COVID-19 worries on MWB, and adding this interaction term to the model explained an additional 7% of variance in MWB, above and beyond the main effects. To probe the significant interaction, simple slopes are displayed in Figure 1. For those low in resilience, there was an inverse association between COVID-19 worries and MWB (shown in the blue dotted line) indicating that increased COVID-19 worries were related to lower MWB, though this simple slope was not significant. Conversely, for those high in resilience, there was a significant *positive* association between COVID-19 worries and MWB (shown in the green solid line) indicating that those who reported more COVID-19 worries indeed reported greater MWB.

**Table 2:** Multiple regression models estimating associations between COVID anxiety, resilience, and flourishing.

	**Model 1**		**Model 2**		**Model 3**	
	***b* (SE)**	***p*-value**	***b* (SE)**	***p*-value**	***b* (SE)**	***p*-value**
Intercept	8.99	**0.002**	7.54	**0.007**	11.20	<0.001
Age	−0.17	0.294	−0.23	0.132	−0.23	0.123
Sex (0 = Female; 1 = Male)	0.25	0.231	0.12	0.534	0.19	0.313
Race (0=White, 1=Other)	−0.22	0.331	−0.33	0.128	−0.41	0.051
Year in School	0.33	**0.034**	0.35	**0.018**	0.30	**0.037**
COVID-19 Anxiety	−0.22	0.055	0.03	0.807	−1.22	**0.006**
Resilience			0.69	**<0.001**	−0.60	0.190
COVID-19 Anxiety × Resilience					0.46	**0.003**
*F*-Statistic	3.07	0.013	5.66	<0.001	6.63	<0.001
*R^2^*	0.14		0.29		0.36	

*Model 1 estimates the main effect of COVID-19 worries on flourishing, Model 2 includes the effect of resilience, and Model 3 estimates the interaction between COVID-19 worries and resilience on flourishing. N = 91. Bolded values represent significance*.

## Discussion

The purpose of the current study was to extend our understanding of COVID-19 worries on student-athletes' MWB by examining potential protective effects of resilience. Study findings only provided partial support of the first hypothesis that COVID-19 worries would be negatively associated with MWB; despite a significant correlation, the effect in the regression analysis narrowly missed the traditional statistical significance threshold when worries were measured on their own. In the final model, worries were once again significant. This nevertheless aligns with the results from several other COVID-19 era studies uncovering an inverse relationship between fear and worries of the virus and mental health and MWB (e.g., Bullard, 2020; Sanborn et al., 2021; Shah et al., 2022). Making direct comparisons between the current studies and other studies is challenging because of the myriad ways that COVID-19 concerns have been measured. The most direct comparison is with another study of college student athletes that used the same worry measure (Graupensperger et al., 2020). Those authors collected data in April 2020 and the mean COVID-19 worries score in their sample was 0.66 higher than ours (on a 5-point scale). This provides support that over time, COVID-19 specific worries amongst student-athletes are decreasing (National Collegiate Athletic Association., 2021). However, the varying significant relationships with MWB in this study indicates that despite the beginning of more widespread vaccine distribution in the United States that was occurring during the time of data collection, student-athletes were still experiencing concerns that could have influenced their overall MWB.

We also found that resilience was positively correlated to and predictive of MWB, supporting our second hypothesis. This confirms the results of several other studies indicating that such a relationship exists (e.g., Killgore et al., 2020; Knowles et al., 2021; Paredes et al., 2021). Resilience is often described as the ability to rebound from a stressor or adversity (Sarkar and Fletcher, 2014; Den Hartigh et al., 2022), which should theoretically help individuals flourish. The pandemic was unprecedented for most people living through it, and therefore grounded in uncertainty. Therefore, individuals with an ability to respond to these changes would be able to maintain their MWB better than those who cannot, a process resilience facilitates. One group of elite athletes described their emerging resilience through the beginning stages of the pandemic by explaining how their reframing of the situation, appraising the situation as a challenge, and accepting how unnatural the event was in their lives helped them cope with their stress (Gupta and McCarthy, 2021). Although it is impossible to know what our sample participants experienced due to the quantitative nature of the study, Gupta and McCarthy's (2021) qualitative research suggests that individuals with higher levels of resilience may have been able to engage in some of these processes that could have led to the positive association with MWB.

The most unique contribution of our study was the moderating role of resilience on MWB. The results supported our third hypothesis that resilience would buffer the relationship between COVID-19 worries and MWB. This finding supports previous research indicating that resilience buffered the relationship between various negative pandemic related constructs and MWB (e.g., Killgore et al., 2020; Chan et al., 2021; Paredes et al., 2021; Shah et al., 2022). The implication is that for those with higher levels of resilience, the possible effects of COVID-19 worries are abated.

Despite our hypothesis that this would occur, the strength and direction of the relationship between high resilience and MWB was somewhat unexpected. Specifically, there was a trend toward significance that for individuals with lower resilience, their MWB decreased as their worry increased. For those with high levels of resilience, their MWB actually *increased* as their COVID-19 worries increased. Although this finding seems somewhat counterintuitive, the current body of research on resilience in the pandemic offers some clues.

The difference in MWB outcomes for those with high vs. low resilience throughout the course of the pandemic has been somewhat drastic (e.g., Paredes et al., 2021; Sampogna et al., 2021). For example, the relationship between stressors and depression was eliminated under conditions of high resilience in one study (Chan et al., 2021). In another sample, the differences between low and highly resilient individuals under low COVID-19 threat was minimal. Under high threat, however, more resilient individuals reported much higher subjective MWB (Paredes et al., 2021). Our findings follow a similar trend. In both scenarios, individuals who reported being highly resilient reported greater levels of MWB under conditions of higher concerns about COVID-19.

One explanation for these findings could be the connection between coping behaviors and other traits often associated with resilience. For example, in a large sample of Italian participants, individuals with low resilience reported more maladaptive coping mechanisms than people with high resilience (Sampogna et al., 2021). This could partly explain why the participants in our sample had higher MWB even with more concerns- they utilized more adaptive coping strategies to mitigate the stress they were experiencing. Taken together with how a challenge mindset is often adopted by resilient individuals (Fletcher and Sarkar, 2016; Gupta and McCarthy, 2021), our participants might have perceived pandemic related stressors and worries as challenges to be overcome.

### Implications

The results of our study suggest that developing resilience in student-athletes could alleviate possible detriments to their MWB associated with the pandemic, which may generalize to other adverse circumstances that place high levels of strain on their lives and athletics. Although one long-term effect of the outbreak might be the growth of resilience (PeConga et al., 2020), it is important to recognize that resilience is not necessarily a natural reaction to stress and adversity. However, resilience is a construct that can be developed (PeConga et al., 2020; Sarkar and Page, 2020), and our results suggest that task is a worthwhile endeavor.

Several authors have outlined suggestions on how to develop resilience (Fletcher and Sarkar, 2016; Sarkar and Page, 2020). These strategies are centered around the idea that resilience is not solely an internal factor, but requires a facilitative environment to grow (Fletcher and Sarkar, 2016; Sarkar and Page, 2020). Therefore, we recommend that athletic departments strive to create environments of high challenge and support in order to aid in the development of resilience in their athletes. There is also preliminary evidence that resilience training programs can improve student-athletes' likelihood of using adaptive coping strategies (Sullivan et al., 2021). Unfortunately, that study did not measure the longitudinal change in resilience as a result of the program, so conclusions cannot be drawn regarding the program's ability to do so. Ultimately, resilience training has clear implications for the overall health and MWB of student-athletes, with an added benefit of possibly improving performance (Sarkar and Fletcher, 2014).

### Limitations and Future Directions

Much of the research on resilience in the pandemic suffers from cross-sectional designs with small sample sizes from single locations (Manchia et al., 2022). Unfortunately, those are also limitations in the current study. Any study taking place at one institution will have restricted generalizability, and this combined with our small sample limits the external validity to other Division III student-athletes. It is possible that a larger sample would have led to stronger effects. Additionally, the cross-sectional nature limits our ability to understand the dynamical changes of resilience, which could have ebbed and flowed throughout the pandemic as a result of not participating in sport or other myriad factors (Knowles et al., 2021; Den Hartigh et al., 2022). No causal connections between resilience and MWB can be inferred from the sample.

Despite these limitations, our study has several strengths. A majority of the currently published research explores the impact of the pandemic immediately in the aftermath of the pandemic declaration, season cancellations, and the onset of lockdowns (Kocjan et al., 2021; Paredes et al., 2021; Sanborn et al., 2021; Shah et al., 2022). A couple studies were conducted in the Fall 2020 semester (National Collegiate Athletic Association., 2021; Valster et al., 2021) and indicated that distress was still high. Given the unique circumstances of the postponement of all fall sports and the beginning of vaccine distributions, the opportunity to get a snapshot of student-athletes' resilience and MWB during the Spring 2021 semester adds to the literature. Another unique contribution of the study lies in the primary result that MWB was higher for individuals with greater resilience despite having high levels of COVID-19 worries.

That main finding opens up several avenues for future research. In general, there is a continued need to monitor the various stressors and responses in resilience research (Den Hartigh et al., 2022). As the pandemic continues on, tracking changes in MWB as a response to the various life stressors individuals experience will strengthen this research. Doing so would require longitudinal designs, which would provide more robust support for the role of resilience as a protective factor for MWB. Additionally, the results indicate a need for in-depth qualitative exploration of individuals who are highly resilient. Specifically, learning more about their process of being resilient through the pandemic would provide important insight as to how they still managed to flourish despite having high worries.

## Conclusion

The results of our study indicate that resilience might be an important protective factor for MWB in collegiate student-athletes during the pandemic. Participants with greater resilience reported higher MWB, even with higher levels of COVID-19 worries. The continued existence of worries during the Spring 2021 semester suggest that athletic departments and stakeholders should still be working to ensure the health and MWB of their student-athletes. Specifically, implementing resilience training programs led by qualified individuals could lead to an overall improvement in the student-athlete experience.

## Data Availability Statement

The raw data supporting the conclusions of this article will be made available by the authors, without undue reservation.

## Ethics Statement

The studies involving human participants were reviewed and approved by Shenandoah University Institutional Review Board. Written informed consent from the participants' legal guardian/next of kin was not required to participate in this study in accordance with the national legislation and the institutional requirements.

## Author Contributions

CW and RH contributed to conception and design of the study. CW collected and organized the data. SG performed the statistical analysis. RH provided supervision over the project. All authors wrote sections of the first draft of the manuscript. All authors contributed revisions, read, and approved the submitted version.

## Conflict of Interest

The authors declare that the research was conducted in the absence of any commercial or financial relationships that could be construed as a potential conflict of interest.

## Publisher's Note

All claims expressed in this article are solely those of the authors and do not necessarily represent those of their affiliated organizations, or those of the publisher, the editors and the reviewers. Any product that may be evaluated in this article, or claim that may be made by its manufacturer, is not guaranteed or endorsed by the publisher.
